# A Prospective Longitudinal Study of Perceived Infant Outcomes at 18–24 Months: Neural and Psychological Correlates of Parental Thoughts and Actions Assessed during the First Month Postpartum

**DOI:** 10.3389/fpsyg.2015.01772

**Published:** 2015-11-20

**Authors:** Pilyoung Kim, Paola Rigo, James F. Leckman, Linda C. Mayes, Pamela M. Cole, Ruth Feldman, James E. Swain

**Affiliations:** ^1^Department of Psychology, University of DenverDenver, CO, USA; ^2^Department of Psychology and Cognitive Science, University of TrentoTrento, Italy; ^3^Child Study Center, Yale University School of MedicineNew Haven, CT, USA; ^4^Department of Psychology, The Pennsylvania State UniversityUniversity Park, PA, USA; ^5^Department of Psychology, Bar-Ilan UniversityRamat Gan, Israel; ^6^Department of Psychiatry, Psychology, Center for Human Growth and Development, Women and Infants Mental Health Program, University of MichiganAnn Arbor, MI, USA

**Keywords:** father, mother, parenting, postpartum, neuroimaging, infant, socioemotional development

## Abstract

The first postpartum months constitute a critical period for parents to establish an emotional bond with their infants. Neural responses to infant-related stimuli have been associated with parental sensitivity. However, the associations among these neural responses, parenting, and later infant outcomes for mothers and fathers are unknown. In the current longitudinal study, we investigated the relationships between parental thoughts/actions and neural activation in mothers and fathers in the neonatal period with infant outcomes at the toddler stage. At the first month postpartum, mothers (*n* = 21) and fathers (*n* = 19) underwent a neuroimaging session during which they listened to their own and unfamiliar baby’s cry. Parenting-related thoughts/behaviors were assessed by interview twice at the first month and 3–4 months postpartum and infants’ socioemotional outcomes were reported by mothers and fathers at 18–24 months postpartum. In mothers, higher levels of anxious thoughts/actions about parenting at the first month postpartum, but not at 3–4 months postpartum, were associated with infant’s low socioemotional competencies at 18–24 months. Anxious thoughts/actions were also associated with heightened responses in the motor cortex and reduced responses in the substantia nigra to own infant cry sounds. On the other hand, in fathers, higher levels of positive perception of being a parent at the first month postpartum, but not at 3–4 months postpartum, were associated with higher infant socioemotional competencies at 18–24 months. Positive thoughts were associated with heightened responses in the auditory cortex and caudate to own infant cry sounds. The current study provides evidence that parental thoughts are related to concurrent neural responses to their infants at the first month postpartum as well as their infant’s future socioemotional outcome at 18–24 months. Parent differences suggest that anxious thoughts in mothers and positive thoughts in fathers may be the targets for parenting-focused interventions very early postpartum.

## Introduction

The first few months after a baby’s birth are a critical period for parents and infants to establish a long-term emotional bond (Winnicott, 1956; Ainsworth, 1979; Porter and Hsu, 2003). Furthermore, the quality of parental care during these months has been established to have an enduring influence on the child’s socioemotional development (Bornstein, 2002; Feldman, 2007a, 2015). Neuroimaging of new mothers and fathers during this critical period suggest that heightened neural sensitivity to infant-related stimuli are associated with parents’ behavioral and emotional sensitivity toward their infants (Barrett and Fleming, 2011; Moses-Kolko et al., 2014; Rilling and Young, 2014; Swain et al., 2014b). These findings indicate the importance of specific aspects of psychological adjustment to parenthood and changes in the parental brain during the early postpartum period because they may contribute to predict later infant outcomes. The current longitudinal study addresses these issues by investigating the relations among postpartum parents’ neural sensitivity to own infant’s cry sounds, their concurrent thoughts and behaviors, and their perceptions of their infants’ subsequent socioemotional outcomes. In addition, this study investigates similarities and differences among these associations in mothers and fathers – thus addressing the paucity of brain research on fathers (Swain et al., 2014a).

During the first few months after birth, infants are sensitive to the quality of parenting and caregiving has long-lasting effects on socioemotional competencies and stress regulation (Essex et al., 2002; Hostinar et al., 2014). Animal models indicate that caregiving quality during the first 10 days leads to epigenetic changes in offspring stress regulation. Offspring who receive low quality maternal care during this very early period of life exhibit increased stress reactivity and anxiety compared to offspring who received high quality maternal care all the way through to adulthood (Meaney, 2010). In humans, maternal sensitivity during the first few months after birth has been associated with infant physical and emotional stress reactivity and emotion regulation several years later (Feldman, 2007a; Lupien et al., 2009; Landers and Sullivan, 2012). Although current research on paternal care is more limited, the quality of father-infant interactions during the first year of the infant’s life has also been associated with emotional and social development in infants, young children, and adolescents (Feldman and Masalha, 2010; Feldman and Bamberger, 2011; Lamb and Lewis, 2013; Ramchandani et al., 2013).

The early postpartum period is also noteworthy for its importance in the establishment of long-term emotional bonds between parents and infants as evident in parent brain physiology. During this period, human mothers and fathers exhibit dynamic neurobiological plasticity (Kim et al., 2010a, 2014, 2015; Pereira and Ferreira, 2015). Voxel-based morphometry analysis reveals that human mothers exhibit structural growth, indicated by increased gray matter volume, in several brain regions, including those involved in maternal motivation and reward processing such as the striatum, amygdala, hypothalamus, and the substantia nigra from the first month to 3–4 months postpartum (Kim et al., 2010a). Structural growth has also been noted in neural areas involved in processing sensory information and empathy, including the superior temporal gyrus, thalamus, insula, and pre- and post-central gyri. Finally, the inferior and medial frontal gyri, as well as the anterior cingulate cortex, regions associated with regulating emotions, also show structural increases. Functional magnetic resonance imaging (fMRI) studies provide converging evidence, with mothers showing increased activations in similar brain regions during the first few months postpartum in response to infant-related stimuli including cry sounds, pictures, and videos (Lorberbaum et al., 2002; Nitschke et al., 2004; Kim et al., 2010b, 2011; Landi et al., 2011; Barrett et al., 2012).

Like mothers, longitudinal changes from the first month to 3–4 months postpartum in human fathers’ brains have been examined using voxel-based morphometry analysis (Kim et al., 2014). Fathers also exhibit anatomical growth in the amygdala and striatum (including putamen and caudate), regions associated with parental motivation, and the lateral prefrontal cortex and insula, regions involved in emotion regulation and social information processing. Recent fMRI studies also suggest that fathers increased brain responses to baby stimuli in these brain regions during the early postpartum period (Atzil et al., 2012; Kuo et al., 2012; Mascaro et al., 2013, 2014; Abraham et al., 2014). For example, during 2–4 months postpartum, fathers also show increased activation in prefrontal and striatal brain regions in response to their own infant images (Kuo et al., 2012).

Thus, the early postpartum period is a sensitive period for parents to undergo neural changes that support close emotional relationships with infants, which then support positive socioemotional development in infants. Moreover, in a published study (Kim et al., 2013), we demonstrated that parenting-related cognition and actions during the transition to parenthood also play a role in sensitive parenting behaviors among mothers and fathers. At the first, then again 3–4 months postpartum, mothers and fathers were asked about their parenting-related thoughts and actions using a semi-structured interview, the Yale Inventory of Parental Thoughts and Actions-Revised (YIPTA-R) (Kim et al., 2013). From the first month to 3–4 months postpartum, both mothers and fathers exhibited a decline in their anxious and intrusive thoughts about parenting and infants, but an increase in their positive thoughts about parenting and infants. Also, at 3–4 months postpartum, higher levels of maternal anxious and intrusive thoughts about parenting and infants were inversely related to sensitivity during interactions with infants. For fathers, higher levels of anxious and intrusive thoughts at 3–4 months postpartum were positively associated with paternal sensitivity, suggesting that their worries and concerns may motivate more involvement, at least in this low-risk sample. Thus, this study provides evidence of links between parenting cognition and parenting behaviors, but little is known about whether parenting-related thoughts can be related to parental neural responses to own infant stimuli during the early postpartum periods, and the implications for infants’ later outcomes.

To address this gap in the literature, the current study recruited new mothers and fathers and interviewed them about their parental thoughts, twice during the early postpartum period - at the first month and three to 4 months postpartum. First, we identified specific parental thoughts that are associated with the infant’s subsequent socioemotional outcomes at 18–24 months postpartum as perceived by their parents. Postpartum negative mood was included as a covariate to examine the unique effects of parenting-related thoughts on infant socioemotional outcome (as reported by the parents), independent of parental mood. Next, we investigated whether parental thoughts at the first vs. later (3–4 months postpartum) time points would better predict their perceptions of their infants’ subsequent socioemotional functioning, as a step in identifying sensitive time windows of parental adjustment and caregiving behavior. These analyses permitted us to focus on specific cognitive aspects of parenting at particular postpartum time points and their associations with positive infant outcomes. In the next step we examined the associations among parental thoughts and parental neural sensitivity to infants. Mothers and fathers participated in an fMRI scanning visit, during which their neural responses to own and unfamiliar infant cry sounds were assessed at the first month postpartum. We examined whether neural responses to own infant cry vs. unfamiliar infant cry sounds were associated with parental thoughts in mothers and fathers. We hypothesized that positive and negative thoughts about parenting and infants would be associated with neural regions particularly involved in reward/motivation and emotion regulation in mothers and fathers. Finally, mediation analyses were conducted to examine the indirect effects of parental thoughts on later infant socioemotional outcomes via parental neural sensitivity to own infant stimuli.

## Materials and Methods

### Participants

Families with infants who were born healthy and full-term were recruited at a Yale-New Haven Hospital postpartum ward. Families first participated in two waves - Time 1 (first month postpartum), and time2 (3–4 months postpartum). The families were re-contacted at Time 3 (18–24 months postpartum). Among families who participated in all three phases, two mothers and two fathers were excluded from the analysis due to excessive motion (>3 mm or degree) during the fMRI session at Time 1.

Thus, a total of 21 mothers (age *M* = 35.56, *SD* = 7.81 at Time 1) and 19 fathers (age *M* = 37.68, *SD* = 4.67 at Time 1) were included in the analyses. Among these parents, 17 mothers and fathers were married to each other. Both mothers (*M* = 17.90 years, *SD* = 3.21) and fathers (*M* = 16.79 years, *SD* = 3.03) were above college educated on average. In the sample, 61.9% of mothers and 47.4% of fathers were first-time parents. All infants of these parents were Caucasian background except one infant whose parents had Caucasian and Hispanic backgrounds and 52.2% of the infants were female. Nine of the 21 mothers and 8 of 19 fathers overlapped with the sample of a previous study (Kim et al., 2013).

### Procedures

A trained researcher visited families’ homes at all three time points, the first month (Time 1), 3–4 months postpartum (Time 2), and 18–24 months postpartum (Time 3). At times 1 and 2, mothers and fathers were interviewed separately by a trained researcher and completed self-report questionnaires. At Time 1, mothers and fathers also underwent a neuroimaging session at a university. At Time 3, mothers and fathers completed questionnaires at home. All procedures were approved by Yale University Human Investigation Committee and patients were fully informed and consented to all procedures.

### Measures

#### Yale Inventory of Parental Thoughts and Actions – Revised (YIPTA-R)

Parenting-related thoughts/behaviors were assessed using YIPTA-R, a semi-structured interview (Leckman et al., 1999; Kim et al., 2013), at times 1 and 2 in mothers and fathers. The six domains of the YIPTA-R were the following: (1) caregiving thoughts and actions about the baby (CARE; e.g., Fed your baby [hrs/per day]); (2) thoughts and actions associated with relationship building (RELATIONSHIP; e.g., Thoughts about baby’s future development); (3) the positive experiences of parenting (POSITIVE PARENTING; e.g., “fulfilling” for describing experience of being a parent); (4) positive thoughts about the baby (POSITIVE BABY; e.g., “perfect” for describing perception of baby); (5) preoccupation regarding the infant’s needs and well-being (PREOCCUPATION; e.g., Mind occupied with thoughts about the baby [hrs/per day in past week]); (6) anxious intrusive thoughts and harm avoidant behaviors (AITHAB; e.g., Worries about something bad happening to the baby, Worries about being up to the task of parenting). More details on items and how the domains were scored are detailed in Leckman et al. (1999) and Kim et al. (2013).

#### The Beck Depression Inventory (BDI)

Beck Depression Inventory (Beck et al., 1988) was used to assess depressive symptoms at all three time points in mothers and fathers. The BDI consists of 21 items, with each item answered on a scale of 0 (symptom is absent) to 3 (symptom is severe).

#### The Spielberger State/TraitAnxiety Inventory (STAI)

State/Trait Anxiety Inventory questions were used to assessstate anxiety levels in mothers and fathers at all three time points (Spielberger and Vagg, 1984). The 20 state anxiety items are rated on a scale of 1 (almost never true) to 4 (almost always true).

#### The Brief Infant Toddler Social Emotional Assessment (BITSEA)

Infants’ socioemotional functioning was assessed using the BITSEA (Briggs-Gowan and Carter, 2002; Briggs-Gowan et al., 2004). Mothers and fathers completed the questionnaires separately at Time 3. This parent report has 42 items, comprising two scales: 11 items assess socioemotional competence and 31 items assess problems. The competencies scale includes items on sustained attention, compliance, mastery motivation, prosocial peer relations, empathy, imitation/play skills, and social relatedness. The problems scale focuses on externalizing, internalizing, and regulatory problems. Parents rate each item on a 3 point scale (0 = not true/rarely, 1 = somewhat true/sometimes, 2 = very true/always). Thus, the range of the scores was 0–33 for competence and 0–93 for problems. The BITSEA has demonstrated construct validity and clinical validity in discriminating children with clinically significant problems from matched control children (Briggs-Gowan et al., 2004; Briggs-Gowan and Carter, 2006; Karabekiroglu et al., 2010). Competence scores below 15th percentile (13 for boys and 15 for girls) or problem scores above 75th percentile (15 for both boys and girls) indicate a possible problem or deficit/delay (Briggs-Gowan et al., 2004). Using the cutoff, based on mother report, one girl in our sample had low competence and one boy had high problems. Based on father report, one girl had low competence, and two boys had high problems.

### fMRI Paradigm

After consenting, parents were asked to record their own infant’s cry samples at home during a diaper change using a portable digital recorder. The control cry samples were selected from cry samples of infants who did not participate in the study (see Kim et al., 2011 for more details). Any non-cry noise and background sounds were removed using sound editing software (Cool Edit Pro Version 2.0, Syntrillium Software, Phoenix, AZ, USA). Using the software, white noise sounds were matched to own baby cry and control cry samples.

Based on the role of baby cry in parental care evocation, especially in the early postpartum (discussed in Swain et al., 2004; Swain and Lorberbaum, 2008), during the fMRI session, mothers and fathers listened through headphones to two types of cry sounds: those of their own baby and of an unknown baby as well as two types of white noise sounds each matched to the infant cries (own noise and control noise). Parents were instructed to attend to and experience naturally the emotional state elicited by each set of sounds. The order of sounds was pseudo-randomized. Each sound was presented for 30 s with 10 s of rest between sounds. There were two runs for each participant and they heard each sound block a total of five times. During the scan, at the end of each sound block, participants rated levels of emotional responses to the sound stimuli by using a button press (1 = none, 2 = a little, 3 = moderate, 4 = maximum emotional response).

### fMRI Data Collection and Processing

Anatomical T1-weighted echo-planar images (spin-echo; TR = 300 ms; TE = 4 ms; matrix size 64 × 64; 30 axial slices; 3.125 mm in-plane resolution, 5 mm thick) were acquired to be coplanar with the functional scans for spatial registration using a Siemens trio 3T full-body scanner (Erlangen, Germany). Then, functional data were acquired (echo planar T2^∗^-weighted gradient-echo, TR = 2000 ms, TE = 30 ms, flip angle = 80°, matrix size 64 × 64, 30 axial slices, 3.125 mm in-plane resolution, 5 mm thick).

Functional imaging data were preprocessed and analyzed using SPM8 (Statistical Parametric Mapping 8; Wellcome Trust Center for Neuroimaging, University College, London, UK; http://www.fil.ion.ucl.ac.uk/spm) and Matlab 7 (The MathWorks, Natick, MA, USA). Two images at the beginning of each fMRI run were discarded to account for magnetic equilibrium. After slice time correction, images within each run were realigned to the third image of the run to correct for movement. After motion correction, the high resolution T1 anatomical images were co-registered to realigned functional images. The high resolution T1 anatomical images were spatially normalized to the SPM8 MNI template using the default setting. The normalized functional images were resampled 2 mm × 2 mm × 2 mm. Images were then spatially smoothed using a Gaussian filter with a full-width half-maximum value of 8 mm.

### Analysis

#### Associations between YIPTA-R and BITSEA

First, a repeated-measure ANOVA was used with parent (mother, father) as a between-subject factor, and time (times 1 and 2) as a within-subject factor to compare YIPTA-R between mothers and fathers across time points. Parental moods (BDI, STAI-state) were compared using a repeated-measure ANOVA with parent (mother, father) as a between-subject factor, and time (times 1, 2, and 3) as a within-subject factor. BITSEA competencies and problems were compared using a one-way ANOVA with parent (mother, father) as a between-subject factor. Next, correlations analyses were performed to examine associations among YIPTA-R, BDI, and STAI-state with the BITSEA variables across time points. Last, stepwise regression analysis was used to identify specific domains of the YIPTA-R that were associated with the BITSEA scores. The stepwise regression accounted for maximal variance in the infant socioemotional outcomes measured by the BITSEA. Two stepwise regression analyses (one to test Time 1 variables, the other to test Time 2 variables) were conducted to predict competencies and problems in infants. The same analyses were repeated in mothers and fathers. The first stepwise regression included six domains of the YIPTA-R (Care, Relationship, Positive Parenting, Positive Baby, Preoccupation, AITHAB) at Time 1 as main predictors, and two parental mood variables (BDI, State Anxiety) at Times 1 and 3, as well as parity (primiparous vs. multiparous) as control variables. The second stepwise regression included six domains of the YIPTA-R at Time 2 as main predictors, and two parental mood variables at times 2 and 3 as well as parity as control variables.

#### fMRI Data Analysis

At the individual subject level, response amplitudes were estimated using the general linear model for each condition using a high pass filter (0.0078 Hz). Conditions included own baby cry, control baby cry, own noise, and control noise. For individual subjects, pair-wise comparison of response amplitudes created contrast images of the blood oxygen level-dependent (BOLD) signal change associated with the three main contrasts, own baby cry minus own noise, control baby cry minus control noise, and own baby cry minus control baby cry. The current analysis was focused on the own baby cry minus control baby cry contrast to identify the relations among parental thoughts/actions and neural responses specific to own baby cry sounds.

For the group-level analysis, contrast images for individual subjects were entered into a random-effects analysis. Multiple regression was performed with the specific YIPTA domain identified from stepwise regression (AITHAB at Time 1 for mothers; Positive Parenting at Time 2 for fathers; see Results) as an independent variable and parity as a covariate of no interest. An initial voxel-wise threshold of *p* < 0.005 and a minimum cluster size of 203 voxels in mothers and 213 voxels for the own baby cry vs. control baby cry contrast gave a corrected *p* < 0.05. This threshold was determined by Monte-Carlo simulations using the 3dClustSim program of the AFNI toolkit. The subcortical regions, including the limbic (hippocampus, parahippocampus, amygdala), striatum, and midbrain regions, are small in structure but are consistently activated in studies of parents (Kim et al., 2010a; Atzil et al., 2011; Barrett and Fleming, 2011; Rilling and Young, 2014; Swain et al., 2014b); thus, a less conservative statistical threshold of *p* < 0.005, uncorrected, with an extent threshold of 10 consecutive voxels was used. Lieberman and Cunningham (2009) have argued that this less conservative threshold still achieves a desirable balance between Types I and II error rates (Lieberman and Cunningham, 2009).

Mediation analyses were performed using PROCESS (Hayes, 2013). The indirect effect of parenting-related thoughts and actions through neural responses to infant cry was tested using 95% bias-corrected Confidence Intervals with bootstrapping procedures (10,000 bootstrap resamples) (Preacher and Hayes, 2008). The 95% bias-corrected Confidence Intervals without the inclusion of 0 indicates a statistically significant indirect relationship, *p* < 0.05 (Preacher and Hayes, 2008).

## Results

### Means and Standard Deviations of Variables

**Table 1** presents the means, standard deviations, and *F* statistics for parental thoughts/actions from the YIPTA-R domains at times 1 and 2, parental depressive and anxious moods at times 1, 2, and 3, as well as infant competencies and problems at Time 3 as reported by mothers and fathers.

**Table 1:** Yale Interview of Parental Thoughts and Actions-Revised (YIPTA-R), parental mood and anxiety at Time 1 (first month) and Time 2 (3–4 months), and infant outcome at Time 3 (18–24 months) with comparison between and time points and parental sex.

	Time 1				Time 2				Time 3			
	Mothers		Fathers		Mothers		Fathers		Mothers		Fathers		Time	Parent
	*M*	*SE*	*M*	*SE*	*M*	*SE*	*M*	*SE*	*M*	*SE*	*M*	*SE*	*F*	*F*
YIPTA-R														
Care	41.37	2.82	19.54	2.18	33.71	2.26	17.41	1.84	–	–	–	–	6.92^∗^	48.75^∗∗∗^
Relationship	7.05	0.46	6.58	0.68	7.29	0.43	6.58	0.43	–	–	–	–	0.10	0.96
Positive parenting	8.29	1.62	4.21	0.60	8.29	1.21	5.89	0.82	–	–	–	–	1.54	4.61^∗^
Positive baby	5.86	0.60	4.21	0.32	6.71	0.65	4.89	0.49	–	–	–	–	3.84	6.93^∗^
Preoccupation	23.95	2.01	17.08	2.27	20.50	1.65	13.39	1.95	–	–	–	–	7.90^∗∗^	7.92^∗∗^
AITHAB	1.03	0.07	0.85	0.06	0.92	0.05	0.77	0.06	–	–	–	–	6.66^∗^	4.30^∗^
BDI	5.60	0.79	5.22	1.88	5.17	1.02	3.50	1.57	4.95	1.20	4.84	1.46	1.43	0.29
STAI – State	31.48	1.71	32.26	2.04	30.20	1.78	31.06	2.16	31.33	2.36	32.05	2.41	0.65	0.10
BITSEA														
Competencies	–	–	–	–	–	–	–	–	18.71	0.38	17.84	0.47	–	2.11
Problems	–	–	–	–	–	–	–	–	8.57	1.32	9.26	1.19	–	0.15

*^∗^p < 0.05, ^∗∗^p < 0.01, ^∗∗∗^p < 0.001.*

Across times 1 and 2, mothers exhibited higher levels of all domains of YIPTA-R except the Relationship domain compared to fathers. At Time 1, mothers and fathers exhibited higher levels of Care, Preoccupation, and AITHAB compared to Time 2. There were no differences in depressive and anxious moods among mothers and fathers at any of the three time points. No difference was found in ratings of infant competencies and problems at Time 3 among mothers and fathers. No interaction between parent and time for the YIPTA-R domains and mood variables was found.

### Parental Thoughts/Actions and Infant Outcomes

Correlation analyses were performed to examine associations among YIPTA-R domains, BDI and STAI-state scores with BITSEA variables at Time 3. In mothers, the AITHAB domains of the YIPTA-R at Time 1 was associated with both BITSEA socioemotional competencies inversely and problems at Time 3, *rs*(21) = -0.53 and 0.61, respectively, *p*s < 0.05. The Relationship domain of the YIPTA-R at Time 1 was also associated with BITSEA socioemotional competencies at Time 3, *r*(21) = -0.49, *p* < 0.05. Both BDI and STAI-state levels at times 1 and 3 were positively associated with BITSEA socioemotional problems at Time 3, *rs*(21) > 0.50, *p*s < 0.05. In fathers, only the Positive Parenting domain of the YIPTA-R at Time 1 was positively associated with the BITSEA socioemotional competencies at Time 3, *r*(19) = 0.51, *p* < 0.05. No other times 1 or 2 domains of the YIPTA-R or mood symptoms were associated with the BITSEA variables in mothers or fathers. The sex of infants was not associated with the YIPTA-R, BDI, STAI-state, and BITSEA scores reported either in mothers and fathers, with one exception. The Positive Baby domain of the YIPTA-R reported by fathers at Time 1 was also associated with the sex of the infants, *r*(19) = 0.55, *p* < 0.05, suggesting that fathers reported higher levels of Positive Baby (positive thoughts about baby) if their babies were females compared to males.

Stepwise regression was conducted to model relations between parental thoughts/actions and infant outcomes. For mothers the full model included six YIPTA-R Time 1 domains, depressive and anxious moods at times 1 and 3, and parity – thus controlling for multiple comparisons. There were two significant predictors of infant outcomes. First, only the AITHAB at Time 1 accounted for variance in infant socioemotional functioning at Time 3, β = -0.53, *p* < 0.05. Lower AITHAB score at Time 1 was associated with higher BITSEA socioemotional competencies at Time 3. Second, for infant problems, only maternal STAI-state levels at Time 3 was significant, β = 0.73, *p* < 0.001. No Time 2 parenting scores predicted either infant outcome.

The same model was run for fathers. The only significant predictor of infant outcomes was in the model predicting socioemotional competencies; higher Positive Parenting accounted better competencies in infants, β = 0.51, *p* < 0.05. No other Time 1 variables at predicted infant problems. Moreover, no Time 2 variables were associated with either infant competencies or problems. Based on these results, only Time 1 AITHAB at Time 1 for mothers and Time 1 Positive Parenting for fathers were used in analyses with neuroimaging data.

### Neural Responses to Own Baby Cry and Parental Thoughts/Actions

**FIGURE 1:**
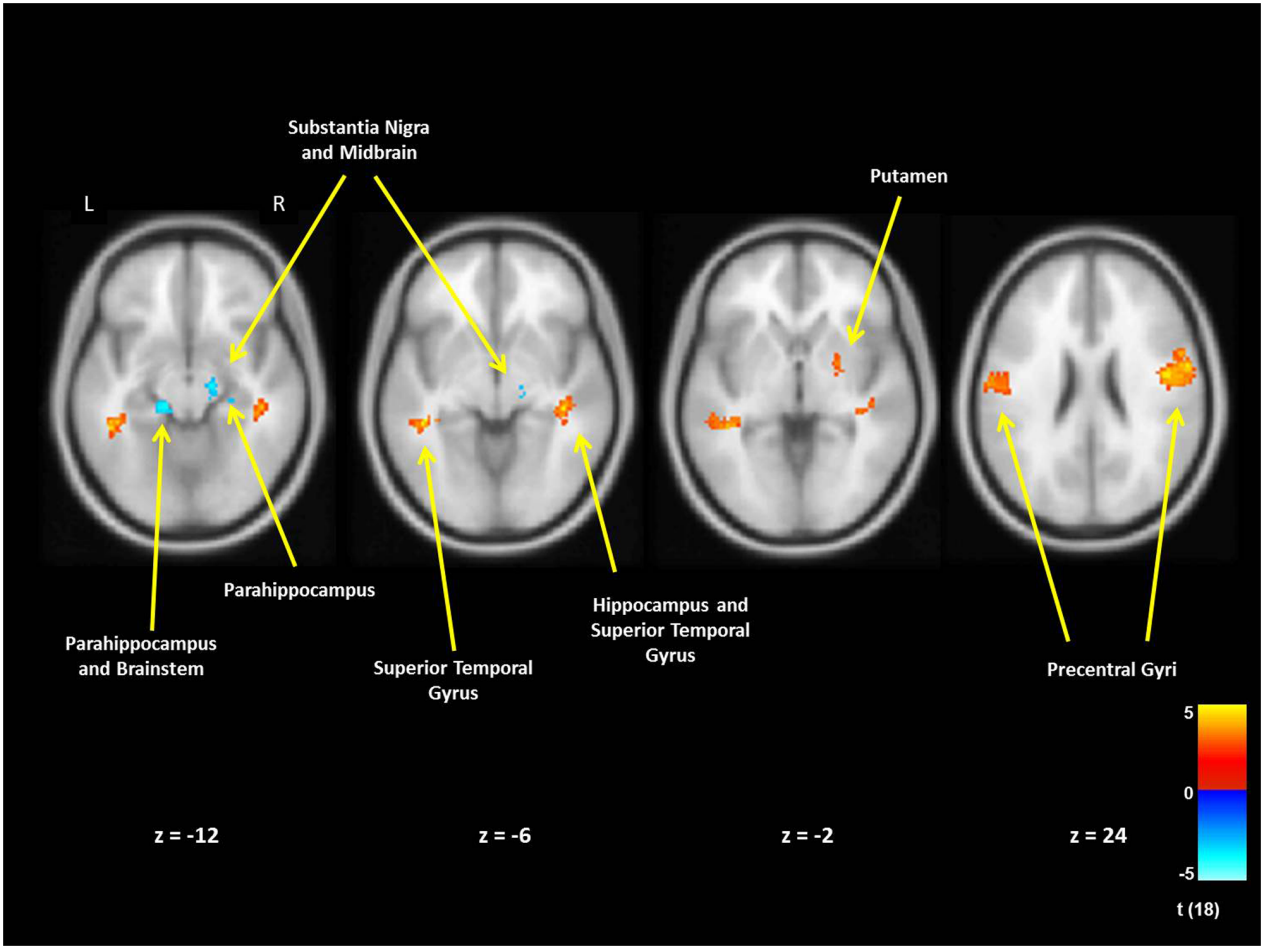
**New mother brain areas with the associations between the AITHAB at Time 1 (first month postpartum) and neural activity in the own infant cry vs. control infant cry contrast at Time 1.** For cortex we set thresholds at *p* < 0.05 (corrected), >203 voxels and for subcortical regions at *p* < 0.005 (uncorrected), >10 voxels.

**FIGURE 2:**
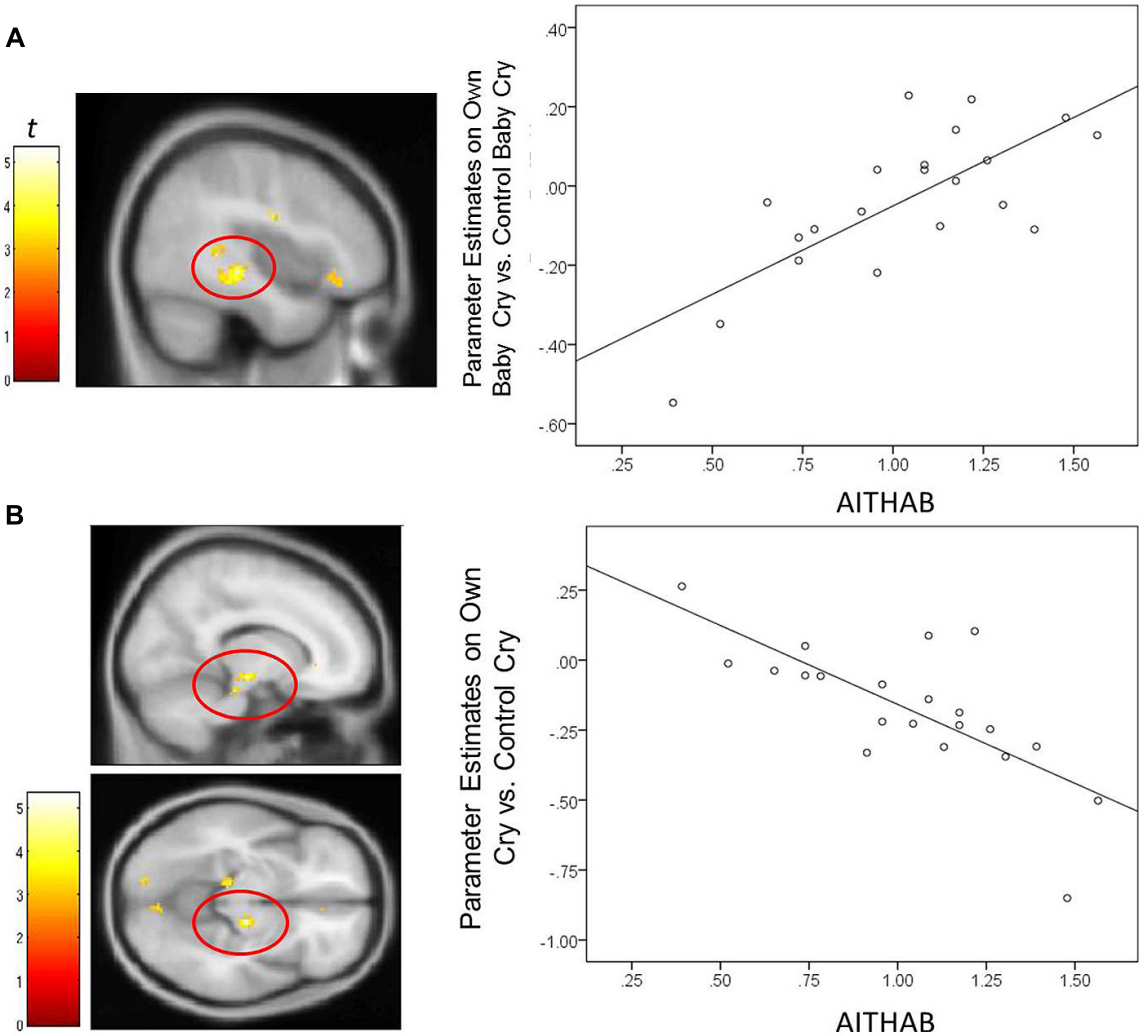
**For new mothers, scatter plots describing the associations between the AITHAB and parameter estimates of a region in the contrast of the own infant cry vs. control infant cry contrast at Time 1. (A)** The right hippocampus (*x*, *y*, *z* = 42, -30, -6; 145 voxels; *p* < 0.005, uncorrected, >10 voxels); **(B)** the right substantia nigra (*x*, *y*, *z* = 14, -18, -10; 68 voxels; *p* < 0.005, uncorrected, >10 voxels).

For mothers and fathers whole brain analyses were conducted to examine associations with the parenting characteristic that had predicted infant outcomes. For mothers, whole brain analysis was used to examine the associations between the AITHAB and neural activation related to the contrast of own baby cry sounds vs. control baby cry sounds at Time 1, controlling for parity (**Table 2**, **Figure 1**). Several cortical regions were positively associated with the AITHAB at *p* < 0.05 corrected: the left superior temporal gyrus and bilateral precentral and postcentral gyri, which are both involved in sensorimotor and social information processing. In the subcortical regions, right hippocampus (**Figure 2A**) and putamen activity were positively associated with the AITHAB, *p* < 0.005, uncorrected. Thus, mothers with higher AITHAB levels exhibited greater responses to own baby cry sounds (vs. control baby cry sounds) in these brain regions. On the other hand, in the subcortical structures, the right substantia nigra (a key reward/motivation region; **Figure 2B**) and bilateral parahippocampi were negatively associated with the AITHAB. Thus, mothers with higher AITHAB scores exhibited reduced neural responses to own infant cry sounds (vs. control cry sounds) in these areas.

**Table 2:** Maternal brain areas with the associations between anxious intrusive thoughts and harm avoidant behaviors (AITHAB) at Time 1 (first month postpartum) and neural activity for own infant cry vs. control infant cry at Time 1 (new mothers).

			MNI coordinates (peak within a cluster)				
Regions	BA	Side	*x*	*y*	*z*	Cluster size	*t*-value
**Cortical structures**
Superior temporal gyrus	41	L	–44	-38	-6	293	5.4ˆ*
Precentral, postcentral gyri	4/43	R	46	-8	24	514	4.96ˆ*
Precentral, postcentral gyri	4/43	L	–50	-10	40	607	4.89ˆ*
**Subcortical structures**
Hippocampus, superior temporal gyrus	41	R	42	-30	-6	145	4.85†
Putamen		R	24	-2	-2	28	3.56†
Parahippocampus		R	26	-26	-16	35	-4.08†
Parahippocampus brainstem		L	-12	-30	-12	38	-4.11†
Substantia nigra, midbrain		R	14	-18	-10	68	-5.29†

*^∗^p < 0.05 (corrected) >203 voxels, †p < 0.005 (uncorrected) >10 voxels.*

**FIGURE 3:**
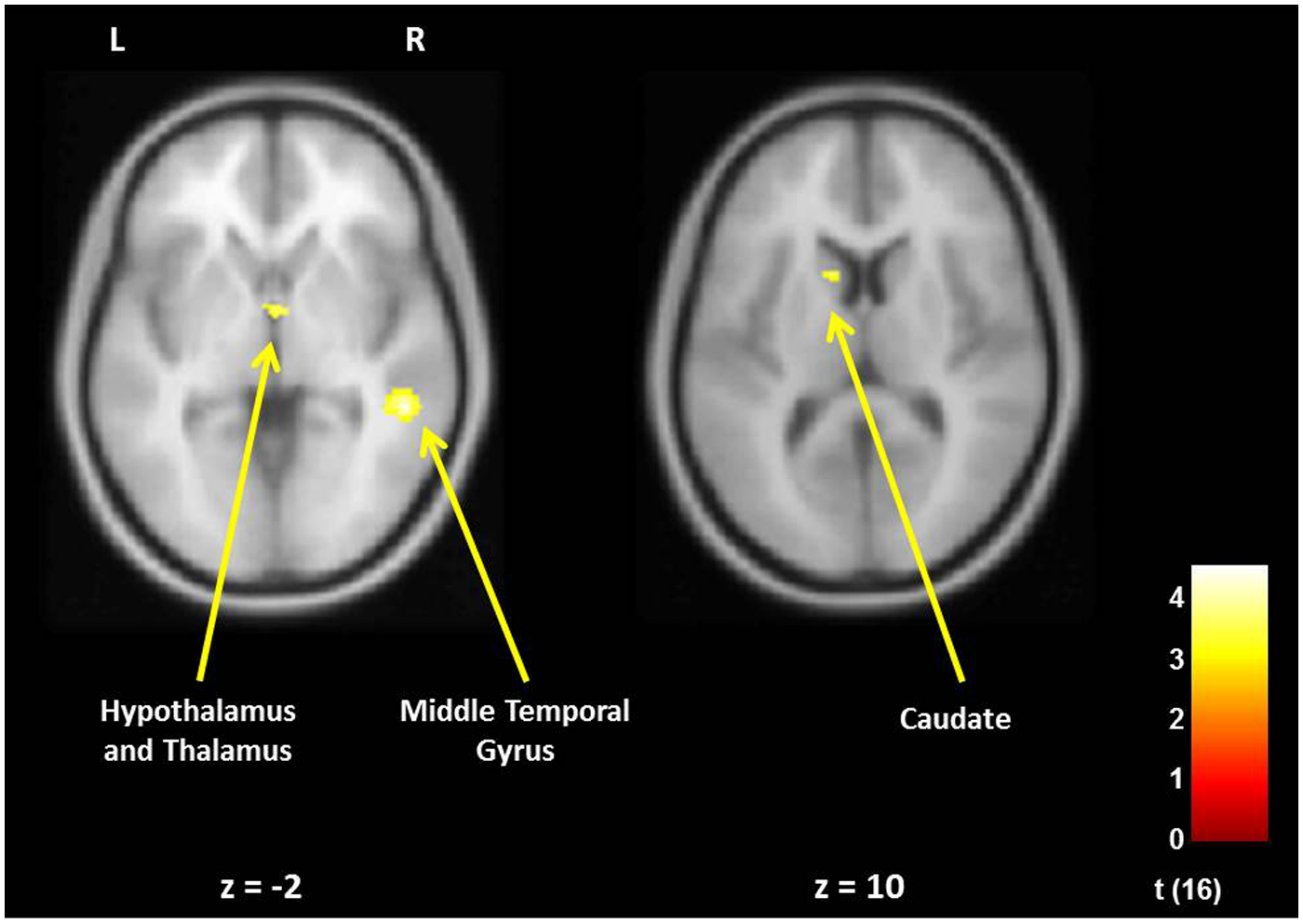
**For new fathers, brain areas with the associations between the Positive Parenting at Time 1 (first month postpartum) and neural activity in the own infant cry vs. control infant cry contrast at Time 1.** Cortical Structure, *p* < 0.05 (corrected), >213 voxels; Subcortical Structure, *p* < 0.005 (uncorrected), >10 voxels.

**FIGURE 4:**
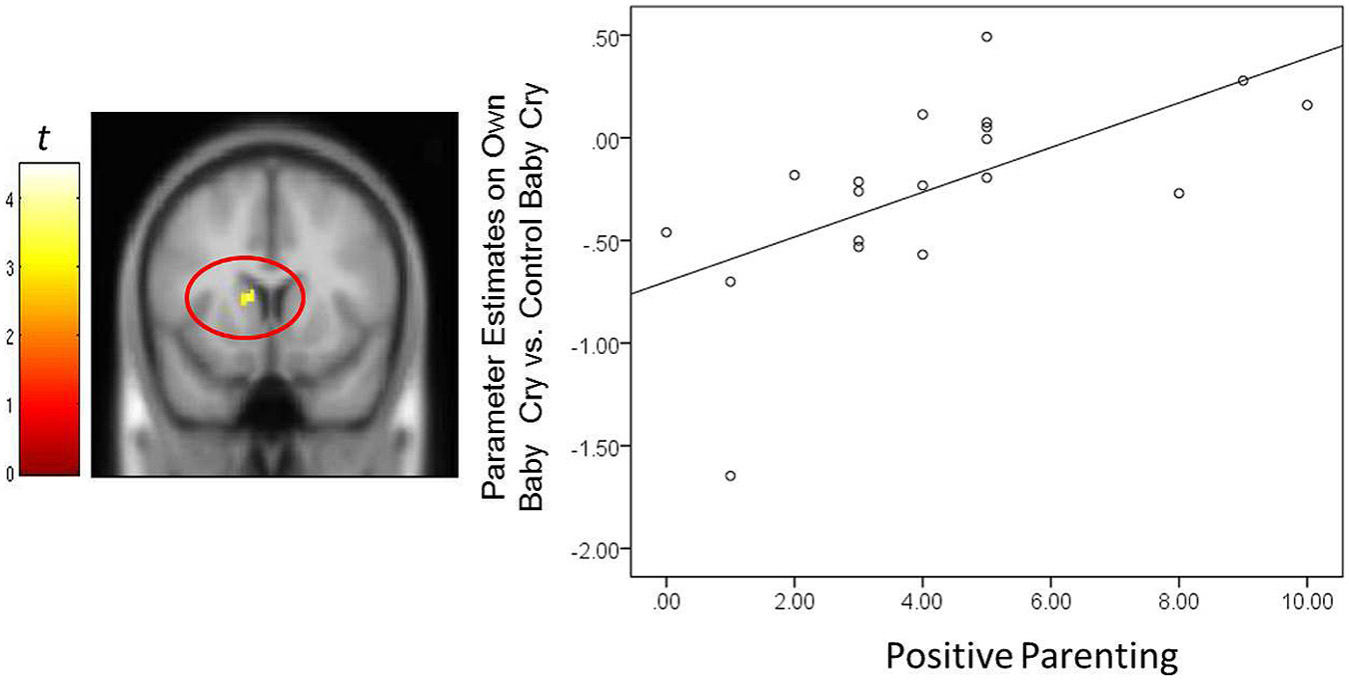
**For new fathers, scatter plots describing the associations between the Positive Parenting and parameter estimates of the left caudate (*x, y, z* = -12, 12, 10; 12 voxels; *p* < 0.005, uncorrected) in the contrast of the own infant cry vs. control infant cry contrast at Time 1**.

In fathers, the whole-brain analysis examined associations between Positive Parenting and neural responses to own baby cry sounds vs. control baby cry sounds at Time 1, controlling for parity (**Table 3**, **Figure 3**). The only cortical region that was positively associated with Positive Parenting was the right middle temporal gyrus (the auditory cortex), *p* < 0.05, corrected. In the subcortical structure, activations in a region including the thalamus and hypothalamus, and the left caudate (**Figure 4**) were also positively associated with Positive Parenting for the contrast of own baby cry sound (vs. control baby cry sound), *p* < 0.005, uncorrected. Thus, fathers with higher levels of positive thoughts about parenting exhibited greater neural responses to own infant cry sounds compared to control cry sounds in the auditory cortex, thalamus/hypothalamus, and caudate.

**Table 3:** Paternal brain areas with the associations between positive parenting at Time 1 (first month postpartum) and neural activity for own infant cry vs. control infant cry at Time 1 (new fathers).

			MNI coordinates(peak within a cluster)				
Regions	BA	Side	*x*	*y*	*z*	Cluster size	*t*-value
**Cortical structures**							
Middle temporal gyrus	21	R	52	-40	-2	224	4.5^∗^
**Subcortical structures**							
Thalamus, hypothalamus		R	4	-2	-2	25	3.56†
Caudate		L	-12	12	10	12	3.56†

*^∗^p < 0.05 (corrected) >213 voxels, †p < 0.005 (uncorrected) >10 voxels.*

Additionally, we conducted the whole-brain analyses examining neural responses to own baby cry sounds between mothers and fathers. We also conducted the whole-brain analyses examining associations with the Positive Parenting in mothers and the AITHAB in fathers (Supplementary Tables S1 and S2). The results suggest that the Positive Parenting and AITHAB were uniquely associated with different neural regions in mothers and fathers.

### Mediating Role of Neural Responses to Infant Cry in the Links between Parental Thoughts/Actions and Parent’s Subsequent Appraisal of Later Infant Socioemotional Outcome

In mothers, the indirect effect of the AITHAB on the BITSEA competence scores through activations of suprathreshold clusters (see **Table 2**) was tested. An indirect effect through the right substantia nigra/midbrain was significant: 2.15, 95% CIs [0.53–4.66]. The higher parenting-related anxious thoughts and actions were associated with reduced substantia nigra/midbrain responses to own infant cry (vs. unfamiliar infant cry) at Time 1. The reduced substantia nigra/midbrain responses were in turn associated with lower socioemotional competence in infants at Time 3. The direct association between the AITHAB and BITSEA score remained significant: -5.12, 95% CIs [-8.23 – -2.02]. Thus, substantia nigra/midbrain responses to own infant cry partially mediated the relation between parenting-related anxious thoughts and actions and infant outcomes in mothers. The indirect effect was not detected in other neural regions in mothers.

In fathers, the indirect effect of Positive Parenting on the BITSEA competence scores through activations of suprathreshold clusters (see **Table 3**) was tested. An indirect effect through the right thalamus/hypothalamus was significant: 0.27, 95% CIs [0.01–0.58]. The higher parenting-related positive thoughts and actions were associated with increased thalamus/hypothalamus to own infant cry (vs. unfamiliar infant cry) at Time 1. The increased thalamus/hypothalamus responses were in turn associated with higher socioemotional competence in infants at Time 3. The direct association between the Positive Parenting and BITSEA score was no longer significant: 0.14, 95% CIs [-0.29 – 0.57]. Thus, thalamus/hypothalamus responses in fathers to own infant cry fully mediated the relation between parenting-related positive thoughts and actions and infant outcomes. The indirect effect was not detected in other neural regions.

## Discussion

Although it is an important developmental transition for a family, little is known about the neurobiological, cognitive and emotional reactions couples experience as they first encounter their infant’s distress, and the implications of their reactions for infants’ later outcomes. The findings from the current study provide evidence of (1) associations between new parents’ parenting-related thoughts and actions and their neural activity when hearing their infant’s cry sounds during the first month postpartum, and (2) associations between those thoughts and actions and the offspring’s later socioemotional competence at 18–24 months. Notably, these associations were found immediately after the infant’s birth and were no longer observed at 3–4 months postpartum. Furthermore, these relations differed for mothers and fathers. Specifically, mothers who reported fewer anxious thoughts about their parenting and baby, and the fathers who reported more positive thoughts about parenting, the better the child’s perceived socioemotional functioning in toddlerhood.

In new mothers, anxious thoughts and actions about parenting and infants (anxious intrusive thoughts and harm avoidant behaviors; AITHAB) in the first month postpartum were associated with infants’ socioemotional competencies at 18–24 months; caring and positive thoughts/actions were not. This finding adds to prior research from this same sample showing that greater parenting-related anxiety was associated with less maternal sensitivity (Kim et al., 2013). Together, these two sets of finding imply that more maternal anxiety at the first month postpartum may interfere with responding sensitively to infants at 3–4 months postpartum. Thus, increased levels of parental anxious thoughts behaviors at the first month postpartum – for mothers – may disrupt the expression of sensitive behavioral responses to infants at 3–4 months postpartum. It is interesting that global maternal mood and anxiety scales did not have similar associations. Perhaps during the early months postpartum, AITHAB, rather than mood and anxiety measures, was associated with infant socioemotional outcome precisely because it is specific to each mother–child dyad. Such anxious thoughts and actions about infant and parenting may diminish the sensitivity of maternal behaviors as a mechanism for later adverse toddler outcome (Haley and Stansbury, 2003; Feldman, 2007b; Feldman et al., 2009).

In mothers, parenting-related anxious thoughts and behaviors during the first month were inversely associated with neural responses in the substantia nigra, a key reward and motivation region. This is in accord with reports of substantia nigra activation specifically in response to own infant-related visual stimuli (Noriuchi et al., 2008; Strathearn et al., 2008). The negative associations we report between anxious thoughts/actions and substantia nigra activity may reveal one of the mechanisms through which new mothers with high infant related anxiety may have a diminished capacity to respond with sensitivity to their infants. The mediation results further support the importance of low substantia nigra activation for links between mothers’ early parenting-related anxious and intrusive thoughts and behaviors, and infants’ lower socioemotional competence reported by parents.

On the other hand, higher levels of parenting-related anxious thoughts and behaviors during the first months were associated with increased neural responses to own infants in several brain regions involved in stress regulation and motor responses, specifically the premotor cortex and fusiform gyrus, as well as the hippocampus. The hippocampus is rich in glucocorticoid receptors and shows increased activation when an individual is exposed to stress (Dedovic et al., 2009). Thus, the increased hippocampal activation among mothers who reported higher anxious thoughts/actions may reflect neural processes of anxious responses to infant cry sounds. Increased responses in the precentral and postcentral gyri and fusiform gyrus, regions for motor responses and face information processing, suggest action-oriented neural responses. The action-oriented neural responses associated with higher anxious thoughts and behaviors were associated with low maternal sychrony observed during interactions with infants (Atzil et al., 2011). The putamen is another interesting region to be associated with AITHAB because its association with the anxiety of obsessive-compulsive disorder (OCD; Huyser et al., 2009; Figee et al., 2013). AITHAB has also been discussed as a potentially adaptive form of subclinical anxiety akin to OCD for new mothers to manifest heightened vigilance for potential threats to the baby, coupled with near-compulsive behaviors that may ensure infant well-being (Leckman et al., 2004). However, high levels of maternal anxiety may diminish the mother’s sensitivity to her infant.

In fathers, we also found that parental thoughts/actions in the first month, but not 3–4 months postpartum, were significantly associated with infant socioemotional competencies at 18–24 months. However, the only domain in fathers that was significantly associated with infant outcomes was different from the one identified in mothers. In fathers, it was the positive perception of being a parent, rather than lower anxious thoughts/actions that was associated with better infant socioemotional outcomes at 18–24 months. This interesting difference between mothers and fathers may be associated with the sex differences in parenting quality that contribute to interactions with infants (Volling et al., 2002). The father–infant interaction style that is associated with positive infant outcomes is characterized by high-intensity positive interactions (e.g., joy, stimulatory play), primarily through physical and sensory stimulations. Researchers suggest that parent–child interactions serve different roles in that while mothers provide emotional comfort and security in response to a child’s distress, fathers provide challenges and encourage explorations during interactions with their infants (Grossmann et al., 2002, 2008). Thus, while anxious thoughts may importantly contribute to reduced ability to respond sensitively to infants in mothers, in fathers, positive thoughts related to parenting may play the most important role for predicting their positive interactions with infants.

Positive thoughts about parenting may be supported by increased own-baby-cry related neural activity, particularly in the auditory cortex, thalamus/hypothalamus, and the caudate. Increased responses to infant cry sounds in the auditory cortex and the thalamus suggest enhanced sensory information processing and integration, as in other studies where increased auditory cortex and thalamus structure and activations were observed in response to baby cry sounds in fathers (Atzil et al., 2012; Kuo et al., 2012; Mascaro et al., 2013, 2014; Kim et al., 2014). The caudate and hypothalamus is part of a parental motivation circuit in animal as well as human models (Numan and Insel, 2003; Montoya et al., 2012; Swain et al., 2012), thus, the increased caudate and hypothalamus activation may also support father’s drive to respond appropriately to their infants, which fits with more positive infant socioemotional outcomes. Increased caudate and hypothalamus structure and activation in response to baby cry have been reported in fathers (Mascaro et al., 2013, 2014; Kim et al., 2014). Furthermore, increased thalamus/hypothalamus response to own baby cry mediated the links between positive thoughts about parenting and better subsequent infant socioemotional outcomes as reported by the parents. Thus, our study provides support that positive thoughts about parenting at the first month postpartum is associated with increased neural responses to infant cry in the thalamus and hypothalamus, possibly for enhanced sensory information processing and parental motivation. Neural responses are then associated with the parental perception of better infant socioemotional competence at 18–24 months.

The current study should be considered in light of limitations. First, because infant outcomes were assessed by parent report, there is a possibility that they were influenced by parents’ mood or cognitive (positive or negative) bias toward their infants. However, studies using the BITSEA to assess infant outcomes have demonstrated that BITSEA scores at age 1–3 prospectively predict teacher-reported behavioral outcomes at age 6, after controlling for parental mood (Briggs-Gowan and Carter, 2008). The BISTEA shows moderate to high correlations with observation ratings of infant outcomes including the Mullen and Vineland Socialization (Karabekiroglu et al., 2010). We would also like to note that, in our study, mother-report and father-report of the BITSEA were similar (**Table 1**) and were correlated [*r*(16) = 0.46, *p* < 0.05] even though levels of thoughts and actions related to parenting were significantly different between mothers and fathers across almost all domains (**Table 1**). Therefore, although not completely independent from self-report bias, it is likely that the BITSEA captured infant outcomes that may be consistent with third-person report or observation ratings. However, it will be important for future studies to include observation ratings or third-person report to examine associations between parental cognition and infant outcomes. Second, data on parental behaviors were available for only a small subset of the sample in this study, and understanding of the role of neural responses to infant cry in linking parenting-related thoughts and parenting behaviors is limited. Our previous work suggested that at 3 months postpartum, anxious and positive parenting-related thoughts were associated with maternal and paternal sensitivity observed during interactions with infants (Kim et al., 2013). Positive parenting behaviors among new mothers were associated with enhanced neural responses to infant stimuli in some regions overlapping with ones identified in the current study - the hippocampus and the parahippocampus (Musser et al., 2012) and the putamen (Wan et al., 2014). Longitudinal studies are needed to further examine how parenting-related thoughts and neural responses to infants at the first month postpartum may be associated with parenting behaviors in both mothers and fathers, which together may further predict infant outcomes at 18–24 months.

Third, for future research, it would be important to consider hormonal measures and their associations with neural and behavioral sensitivity to infants. During the early postpartum period, both mothers and fathers experience changes in levels of hormones, supporting their new roles as parents (Fleming et al., 2002; Gordon et al., 2010; Storey and Walsh, 2013; Swain et al., 2014a). Important hormone-brain systems for future studies may include oxytocin and vasopressin, for which levels have been associated with neural responses to infant stimuli among mothers and fathers (Atzil et al., 2012). Anxiolytic effects of oxytocin have been consistently observed among postpartum mothers (Riem et al., 2011), thus mothers with higher levels of oxytocin may exhibit lower levels of parenting-related anxious thoughts and actions, which may further be associated with neural and behavioral sensitivity to infants. Also, reduced testosterone levels were also associated with increased neural responses to own child’s images in the reward circuits among fathers (Mascaro et al., 2013), thus fathers with lower levels of testosterone may be more likely to report higher levels of the positive thoughts about parenting, which further be associated with more optimal neural and behavioral sensitivity to infants. Furthermore, stress hormone reactivity has been associated with maternal brain connectivity between the hypothalamus and septal regions known to regulate parenting in animal models (Ho et al., 2014). Fourth, the current study’s sample primarily includes participants from middle- to high-SES backgrounds, and the Caucasian population. The fact that the participants were largely from well-supported backgrounds may further be associated with a limited range of later problems reported among their infants. This may explain why our findings were significant only with competencies but not with problems among infants. Thus, future studies should include a larger and more diverse sample to examine the associations among parental brain functioning, psychological adaptations to parenthood, and later infant outcomes. The associations between parental thought/actions and neural responses to infants would also be important to study in parents who are at risk, such as those with psychopathology, substance use, or trauma exposure. Previously, mothers with a history of depression, trauma, or substance use exhibited altered neural responses to baby cry sounds (Landi et al., 2011; Laurent and Ablow, 2011; Schechter et al., 2012), which may be further associated with difficulties in sensitive parenting. Data on specific parenting-related thoughts associated with atypical neural responses to infants can enhance understanding of parents who are at greater risk for difficulties in adjustment to parenthood, as well as infant socioemotional problems.

Two important strengths of this study are the longitudinal design and the inclusion of both mothers and fathers. Studies using prospective and longitudinal designs provide implications for the timing of interventions. We found that the first month, rather than 3–4 months postpartum, was a sensitive period when psychological adjustment to parenthood and parenting-related thoughts/actions in both mothers and fathers had significant associations with infant outcomes a year and half later. Thus, to support optimal parent–infant relationships, it is likely important to support parents’ adjustment to parenthood during or even possibly prior to the first month as future studies might explore. By including both mothers and fathers in one study, which has rarely been done in previous studies, we also identified sex differences in important aspects of parental thoughts and actions that are significant for long-term infant outcomes (Panter-Brick et al., 2014). There is also a potentially generalizable finding that it is the personally specific measures of parenting in addition to global mood and anxiety scales, that were associated with infant outcome underlining the importance of these measures for future studies that undertake to study transgenerational. Furthermore, we speculate that the combinations of personally tailored assessments and brain imaging stimuli may advance the neuroimaging field that has been hampered by inconsistencies using impersonal measures and stimuli to understand parental moods. The findings also have specific implications for the parental brain field given apparently differential targets for mothers and fathers when supporting their transitions to parenthood. Our findings suggest that efforts to reduce specifically anxious thoughts about parenting and baby in mothers, and efforts to increase positive thoughts about parenting in fathers, would be most effective in supporting adjustments to parenthood and improving parent–infant inactions. The neuroimaging evidence provides evidence for specific neural mechanisms underlying how parental thoughts and actions, distinct for mothers and fathers, are associated with positive infant outcomes.

## Conflict of Interest Statement

The authors declare that the research was conducted in the absence of any commercial or financial relationships that could be construed as a potential conflict of interest.
