# Supplementary material for: A Prospective Longitudinal Study of Perceived Infant Outcomes at 18–24 Months: Neural and Psychological Correlates of Parental Thoughts and Actions Assessed during the First Month Postpartum
Source: Front Psychol. 2015 Nov 20;6:1772. doi: 10.3389/fpsyg.2015.01772 (PMC4654106; doi:10.3389/fpsyg.2015.01772)
Supplement: Supplementary file 1 [file Table_1.DOCX]

Supplementary Material

Neural and Psychological Correlates of Parenting in Mothers and Fathers During the First Postpartum Month; Associations with Infant Outcomes at 18-24 months

Pilyoung Kim*, Paola Rigo, James F. Leckman, Linda C. Mayes, Pamela M. Cole, Ruth Feldman, James E. Swain

*** Correspondence:** Pilyoung Kim: pilyoung.kim@du.edu

# Supplementary Data

We conducted the whole-brain level analysis to compare neural responses to own baby cry sounds between mothers and fathers. The results suggested that there was no cluster indicating differences between mothers and fathers at p < 0.05, corrected. In the subcortical regions at *p* < 0.005, uncorrected, > 10 voxels, there was one cluster in left caudate body (x, y, z = -10, 14, 16; 42 voxels; *t* = 3.64) indicating greater responses to own baby cry sounds in mothers compared to fathers. There was no cluster indicating greater responses to own baby cry sounds in fathers compared to mothers. Therefore, overall mothers and fathers exhibited similar neural responses to their own baby sounds.

Next, we conducted two whole-brain level analyses – (1) associations with the Positive Parenting in mothers and (2) associations with the AITHAB in fathers. The results of the analyses are presented in Supplementary Table 1 and 2 below. The results suggest that there were no overlapping regions that were associated with the Positive Parenting between mothers (Supplementary Table 1) and fathers (Table 3 in the main manuscript). Similarly, there were no overlapping regions that were associated with the AITHAB between mothers (Table 2 in the main manuscript) and fathers (Supplementary Table 2). Thus, the results suggest that the associations with AITHAB and Positive Parenting were identified in separate sets of neural regions between mothers and fathers.

# Supplementary Figures and Tables

## Supplementary Tables

| **Regions** |  |  | **MNI coordinates** | | |  |  |
| --- | --- | --- | --- | --- | --- | --- | --- |
|  |  |  | **(peak within a cluster)** | | |  |  |
|  | **BA** | **Side** | **x** | **y** | **z** | **Cluster size** | **t-value** |
|  |  |  |  |  |  |  |  |
| *Cortical Structures* |  |  |  |  |  |  |  |
| Inferior Parietal Cortex, Supramarginal Gyrus, Angular gyrus, Postcentral Gyrus | 2/3/40 | L | -48 | -48 | 40 | 1016 | 7.59* |
| Inferior Frontal Gyrus, Precentral Gyrus | 44/45 | L | -54 | 10 | 10 | 431 | 5.99* |
| Inferior Parietal Cortex, Supramarginal Gyrus, Angular Gyrus | 40 | R | 40 | -40 | 46 | 648 | 5.68* |
| Supplementary Motor Area, Superior Frontal Gyrus, Dorsal Anterior Cingulate Cortex | 6/8/9/32 | L | -6 | 30 | 40 | 483 | 4.94* |
|  |  |  |  |  |  |  |  |
| *Subcortical Structures* |  |  |  |  |  |  |  |
| Lateral/Medial Globus Pallidus |  | R | 18 | -4 | -2 | 14 | 3.45† |
|  |  |  |  |  |  |  |  |

**Supplementary Table 1.** Maternal brain areas with the associations between Positive Parenting at Time 1 (first month postpartum) and neural activity for own infant cry vs. control infant cry at Time 1 (new mothers).

** p < .05 (corrected) >201 voxels, † p < .005 (uncorrected) > 10 voxels*

**Supplementary Table 2.** Paternal brain areas with the associations between Anxious Intrusive Thoughts and Harm Avoidant Behaviors (AITHAB) at Time 1 (first month postpartum) and neural activity for own infant cry vs. control infant cry at Time 1 (new fathers).

| **Regions** |  |  | **MNI coordinates** | | |  |  |
| --- | --- | --- | --- | --- | --- | --- | --- |
|  |  |  | **(peak within a cluster)** | | |  |  |
|  | **BA** | **Side** | **x** | **y** | **z** | **Cluster size** | **t-value** |
|  |  |  |  |  |  |  |  |
| ***Subcortical Structures*** |  |  |  |  |  |  |  |
| Caudate Body |  | R | 10 | 0 | 18 | 101 | 4.21† |
|  |  |  |  |  |  |  |  |

*† p < .005 (uncorrected) > 10 voxels*
